# Laser-Driven Ultrashort Pulsed Electron Beam Radiation at Doses of 0.5 and 1.0 Gy Induces Apoptosis in Human Fibroblasts

**DOI:** 10.3390/ijms20205140

**Published:** 2019-10-17

**Authors:** Nelly Babayan, Bagrat Grigoryan, Lusine Khondkaryan, Gohar Tadevosyan, Natalya Sarkisyan, Ruzanna Grigoryan, Lilit Apresyan, Rouben Aroutiounian, Natalia Vorobyeva, Margarita Pustovalova, Anna Grekhova, Andreyan N. Osipov

**Affiliations:** 1 Institute of Molecular Biology NAS RA, 7 Hasratyan, 0014 Yerevan, Armenia; n_babayan@mb.sci.am (N.B.); l_khondkaryan@mb.sci.am (L.K.); g_tadevosyan@mb.sci.am (G.T.); n_sarkisyan@mb.sci.am (N.S.); r_grigoryan@mb.sci.am (R.G.); apresyanlilit@rambler.ru (L.A.); 2 Yerevan State University, 1 Manoogian, 0025 Yerevan, Armenia; rouben_a@hotmail.com (R.A.); 3 CANDLE Synchrotron Research Institute, 31 Acharyan, 0040 Yerevan, Armenia; grigory@asls.candle.am (B.G.); 4 State Research Center - Burnasyan Federal Medical Biophysical Center of Federal Medical Biological Agency, 46 Zhivopisnaya, 123182 Moscow, Russia; nuv.rad@mail.ru; 5 Semenov Institute of Chemical Physics. Russian Academy of Sciences, 4 Kosygina, Moscow 119991, Russia; 6 Moscow Institute of Physics and Technology, Dolgoprudny, Moscow Region 141700, Russia; pu.margo@mail.ru; 7 Emanuel Institute for Biochemical Physics. Russian Academy of Sciences, Moscow 119991, Russia; annagrekhova1@gmail.com

**Keywords:** ionizing radiation, ultrashort pulsed electron beam, γH2AX, apoptosis, micronuclei

## Abstract

Rapidly evolving laser technologies have led to the development of laser-generated particle accelerators as an alternative to conventional facilities. However, the radiobiological characteristics need to be determined to enhance their applications in biology and medicine. In this study, the radiobiological effects of ultrashort pulsed electron beam (UPEB) and X-ray radiation in human lung fibroblasts (MRC-5 cell line) exposed to doses of 0.1, 0.5, and 1 Gy are compared. The changes of γH2AX foci number as a marker of DNA double-strand breaks (DSBs) were analyzed. In addition, the micronuclei induction and cell death via apoptosis were studied. We found that the biological action of UPEB-radiation compared to X-rays was characterized by significantly slower γH2AX foci elimination (with a dose of 1 Gy) and strong apoptosis induction (with doses of 0.5 and 1.0 Gy), accompanied by a slight increase in micronuclei formation (dose of 1 Gy). Our data suggest that UPEB radiation produces more complex DNA damage than X-ray radiation, leading to cell death rather than cytogenetic disturbance.

## 1. Introduction

During the last decade, the technology of laser-based acceleration has been developed and its biological and medical applications discussed [1,2,3]. Electron accelerators driven by ultrashort laser pulses in the femtosecond to picosecond duration range generate bunches, which are accelerated to energies of from a few MeV up to a few hundreds of MeV, enabling fast delivery of doses to cells [4,5,6,7]. The possibility of creating directed ultra-short pulses of an enormous dose rate (up to kGy/s) may allow for the precise dose control induction of local effects in tissues [3,8]. Considering the duration of laser-generated electron bunch (pulse widths) given above, and comparing it to the time scale of radiobiological effects in cells, it may be possible that due, to the shortness of the pulses, new radiation effects will arise, ultimately resulting in a change in the radiobiological effectiveness [9]. Up until now, the application of sub-picosecond electron beams with the energy in MeV domain has been used to explain the ultrafast elementary events occurring in confined clusters of ionization [10]. Rigaud et al. [11] have discussed the advantages of using ultrashort electron pulses with a high dose rate (10^13^ Gy/s per pulse) for the complete characterization of radiation-induced DNA damage and repair. The changes in biological response, reflected in the micronucleus formation and dependent on the dose rate as a function of pulse width, were shown [12]. Thus, the study of the radiobiological effects determined by the ultrashort pulse width of electron beam irradiation is of great interest.

The latest achievements in laser technology have led to the development of laser-driven linear accelerators providing ultrashort electron pulses (sub-pico or femtosecond) with electron energy in the MeV domain and pulse repetition rates up to GHz [13,14]. In comparison to laser-based accelerators, these facilities produce electron pulses generated by a UV laser and accelerated using a high-gradient RF resonator, enabling us to precisely form the beam parameters and providing the high stability and reproducibility of electron beam and radiation characteristics. So, this new type of laser-driven accelerators provides a unique opportunity to investigate the effects of ultrashort pulsed electron beam (UPEB) irradiation with the energy in MeV domain at different dose rates, providing sufficient beam stability, reproducibility, and reliability for radiobiological experiments. However, an analysis of the basic mechanisms of laser-driven UPEB radiation damage on living cells is still required to enhance its application in medicine.

The aim of the current study was to investigate the radiobiological effects of sub-picosecond UPEB irradiation in vitro, compared to X-ray (reference) irradiation. Since DNA double-strand breaks (DSBs) are the most deleterious lesions induced by ionizing radiation, the phosphorylated histone H2AX, as a marker of DSB formation after irradiation, was studied. Apoptosis/necrosis, as well as micronuclei formation, were also investigated as common endpoints during radiobiological studies. As an experimental model, the MRC-5 cell line was used, which represents well-characterized human normal fibroblasts approved for radiobiological research [15] and is an appropriate model system for studying early and late radiation effects [16,17,18,19].

## 2. Results

### 2.1. γH2AX Foci Analysis

The dose–response and time course experiments of γH2AX foci formation and elimination in the MRC5 cell line after UPEB and X-ray irradiation were carried out covering 0.1–1 Gy doses of irradiation and the time points 1 h, 4 h, and 24 h (1 Gy).

**Figure 1:**
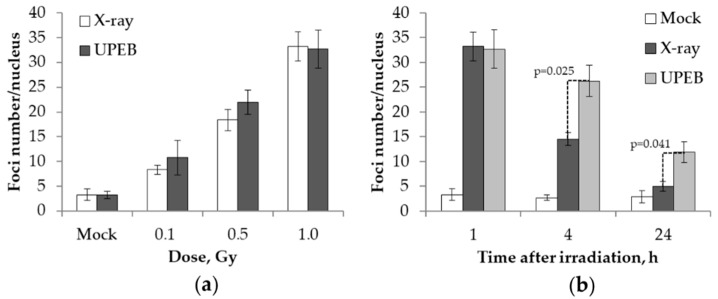
γH2AX foci formation and elimination in the MRC5 cell line after UPEB and X-ray irradiation: (**a**) Dose-dependent (1 h after irradiation) increase in number of foci; (**b**) time-dependent (1 Gy irradiation dose) decrease in number of foci. Data represent the mean ± standard error of the results of three independent experiments.

Based on the linear regression fit, the average yield of γH2AX foci per unit of absorbed dose 1 h after exposure was 27.9 ± 6.0 and 29.0 ± 4.2 foci/cell/Gy for UPEB and X-ray irradiation, respectively (Figure 1a).

**Figure 2:**
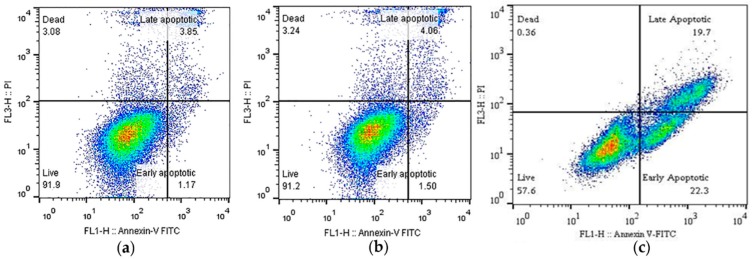
Dot blots of cell viability and apoptosis detection in the MRC5 cell line 24 h after irradiation: (**a**) Mock-irradiated cells; (**b**) cells after X-ray irradiation at a dose of 1 Gy; (**c**) cells after UPEB irradiation at a dose of 1 Gy.

The results of time course experiments, reflecting DNA repair kinetics following UPEB and X-ray irradiation, are presented in Figure 2b. The maximum number of γH2AX (32.7 ± 3.9) foci was observed 1 h post-UPEB irradiation, followed by a 20% decrease in the following 3 h (Figure 1b). The number of γH2AX foci (11.9 ± 2.1) after 24 h of UPEB irradiation was higher than the background level (2.9 ± 1.3), demonstrating the slow repair of DSBs induced by UPEB radiation. Faster elimination of DSBs was observed in the case of X-ray irradiation, with about a 60% decrease in the number of γH2AX foci 4 h post-irradiation (Figure 1b). Residual γH2AX foci were detected after 24 h of X-ray irradiation (5.0 ± 0.9), which was close to the background level (2.9 ± 1.3).

### 2.2. Cell Viability and Apoptosis Analysis

The viability of MRC5 cells, as well as the incidence of apoptotic cell death induced by 0.1 Gy, 0.5 Gy, and 1 Gy doses of UPEB and X-ray radiation, was evaluated using flow cytometric analysis by Annexin V/PI staining 24 h after irradiation. A representative image of flow cytometric analysis after UPEB irradiation at the dose of 1 Gy is given in Figure 2.

**Figure 3:**
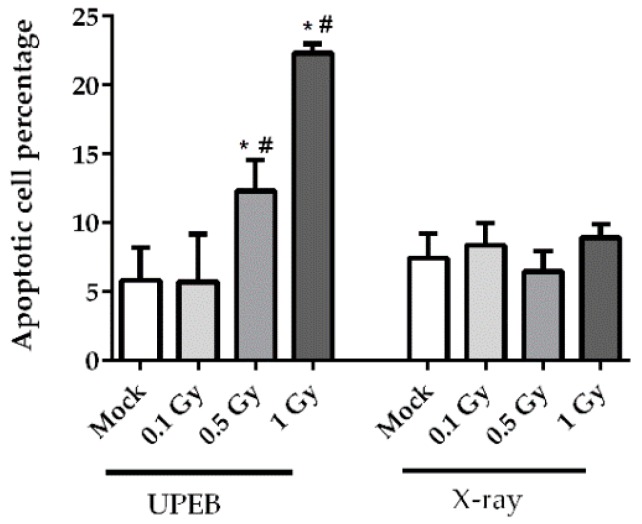
The percentage of apoptotic MRC5 cells 24 h after UPEB and X-ray irradiation. * *p* < 0.05 in comparison to control (mock-irradiated cells). # *p* < 0.05 in comparison to corresponding doses of X-ray irradiation. Data represent the mean ± standard error of the results of three independent experiments.

No significant changes in cell viability were found at any dose of X-ray irradiated cells after 24 h (Figure 3). Similarly, UPEB irradiation at a dose of 0.1 Gy did not affect cell viability. The level of cell viability (88.0 ± 4.9%) and apoptosis (5.7 ± 3.4%) was comparable to the background levels of 87.3 ± 5.4% and 5.8 ± 2.3%, respectively. However, the 0.5 Gy and 1 Gy doses of UPEB irradiation resulted in a significant increase in the percentage of early apoptotic cells at 24 h, as compared to mock-irradiated and corresponding doses of X-ray irradiated cells (Figure 3). At the 0.5 Gy dose of UPEB irradiation, the viability of cells decreased to 76.9 ± 1.2% with 12.7 ± 3.8% apoptotic cells, and at 1 Gy of irradiation the 60.4 ± 3.4% viability was accompanied by 21.9 ± 1.4% apoptotic cells.

### 2.3. Micronuclei Formation

**Figure 4:**
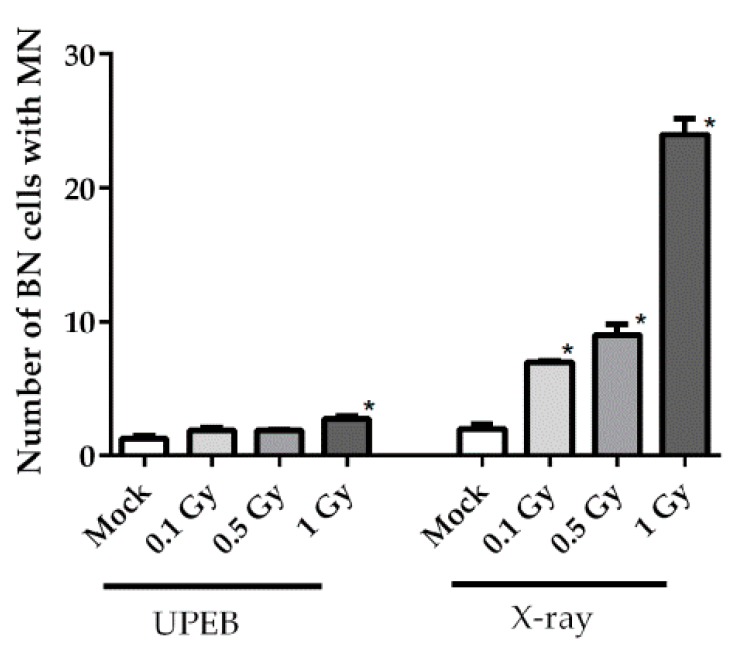
Incidence of micronuclei (MN) formation per 1000 binucleated (BN) cells (‰) in the MRC5 cell line after UPEB and X-ray irradiation. Data represent the mean ± standard error of the results of three independent experiments. * *p* < 0.05 in comparison to the corresponding control (mock-irradiated cells).

The micronuclei frequency was evaluated in the MRC5 cell line after 0.1 Gy, 0.5 Gy, and 1 Gy doses of UPEB and X-ray irradiation. A slight, but statistically significant, increase was observed at 1 Gy of UPEB irradiation (Figure 4); the frequency of cells with MN was 2.8 ± 0.3‰ with a background level of 1.3 ± 0.3‰. The UPEB irradiation of cells at doses of 0.1 Gy and 0.5 Gy did not induce micronuclei in MRC5 cells. A dose-dependent increase in MN frequency was observed after X-ray irradiation, reaching the level of 24.4 ± 2.1‰ of BN cells with MN at the irradiation dose of 1 Gy.

Both UPEB and X-ray radiation produced approximate linear changes in the frequency of micronuclei, with the dose–response function of y = 1.2x + 1.5 (*R*^2^ = 0.83) and y = 20.2x + 2.5 (*R*^2^ = 0.92), respectively.

## 3. Discussion

In this work, the effects of UPEB radiation on DNA damage/repair, cell viability, and micronuclei formation were studied in vitro, and compared with the same endpoints after X-ray (reference) radiation. The level of induced DNA DSBs (γH2AX foci), as well as repair after X-ray irradiation in the MRC5 cell line, shown in this work, corroborates previous results obtained on the same cell line and same radiation type, where an average yield of 36 foci/cell/Gy were reported [20] and 5–10% of residual γH2AX foci was detected after 24 h [21,22]. Later, it was suggested that these residual foci are not DNA double-strand breaks, but indicate an aberrant chromatin structure due to illegitimate rejoining [23]. In the case of UPEB radiation, the average yield of γH2AX foci per unit of absorbed dose was similar to that with X-ray radiation; however, the level of residual foci detected after UPEB irradiation was 4-fold higher, suggesting differences in the activated repair mechanisms and therefore the possibly different nature of the induced DNA damage. The faster elimination of X-ray-induced DSBs shown in our experiments also supports this suggestion, since a 60% decrease in the number of γH2AX foci was observed 4 h post-irradiation, whereas only 20% of UPEB-induced damage was repaired at the same time point. The differences in the repair kinetics are attributed to the level of fast and slow repair components involved in this process, and depend on the complexity of DNA lesions [24]. It is known that there is a higher contribution of the slow component of DNA DSBs repair in the case of more complex DNA damage that includes two or more individual types of lesions within one or two helical turns of the DNA [24] and can be associated not only with DSBs, but also with abasic sites (apurinic/apyrimidinic), damaged bases (oxidized purines or pyrimidines), and single-strand breaks [25]. So, it can be concluded that UPEB-induced DNA DSBs are characterized by slow repair kinetics, suggesting the formation of complex DNA damage.

The knowledge of DNA damage and repair in cells after pulsed electron beam radiation is very limited. The formation of γH2AX after pulsed electron beam irradiation was investigated by Laschinsky et al. [26] and Beyreuther et al. [27] using human normal and cancer cells. Laschinsky et al. [26] reported a low level of γH2AX foci (up to one foci per cell) 24 h post-irradiation at a dose of 1 Gy and pulse duration of 1 × 10^−12^ s (2.4 × 10^9^ Gy/s per pulse; dose rate 0.3 Gy/min), which was comparable to the background level of γH2AX foci (0.4 ± 0.02 foci per cell) in cells. In the case of UPEB irradiation, shown in this study, at the same dose of 1 Gy, but with a shorter pulse duration of 0.4 × 10^−12^ s (1.6 × 10^10^ Gy/s per pulse; dose rate 0.9 Gy/min), a high level of residual γH2AX foci (11.9 ± 2.1 foci per cell) was observed, which was higher than the background level (2.9 ± 1.3). The pulsed electron beam irradiation with longer pulse duration (5 × 10^−12^ s) was used by Beyreuther et al. [27], and a low level of γH2AX foci (up to five foci per cell) was observed after 24 h of irradiation at a dose of 4 Gy (1.6 × 10^8^ Gy/s per pulse; dose rate 0.3 Gy/min). Since a number of studies reported that the dose rate (up to 75 Gy/min) and dose per pulse (up to 7.4 cGy/pulse) do not affect the radiobiological characteristics of the electron beam [26,27,28,29], it can be assumed that the differences observed after UPEB irradiation, reflected in the formation of more complex DNA damage, as characterized by delayed repair [30,31], are attributed to the shorter pulse duration during irradiation. This effect can be explained by the energy deposition during multiple ultrashort pulsed irradiations, which may reduce radical–radical interactions and favor radical–DNA target interactions, thus leading to more complex DNA damage [12].

Radiation-induced cytogenetic abnormalities represent an early marker of possible delayed effects [32,33]. In this work the cytokinesis-block micronucleus (CBMN) assay, which is a valuable biodosimetric tool for quantifying radiation-induced cytogenetic abnormalities [34,35], was used to analyze the genotoxic capacity of UPEB radiation, compared to X-ray radiation. It was reported to be applicable for reliable dose–response estimates up to the 7 Gy of low-LET radiation [36]. Earlier, the dose-dependent increase in X-ray-induced chromosomal damage, such as dicentric chromosomes [37] or micronuclei formation [38], was observed in MRC5 cells at a dose of 0.1–1 Gy, which agreed with our results (the yield of micronuclei after X-ray radiation). A much lower level of micronuclei frequency in the case of UPEB irradiation was shown in our study, compared to X-ray radiation. A significant increase of MN frequency was observed only at the dose of 1 Gy of UPEB radiation, which was around 9-fold lower than that of X-ray radiation. This can be explained by the elimination of cells with highly damaged DNA via apoptosis before cells pass the first cell cycle after irradiation, thus preventing the accumulation of genomic abnormalities in future generations of cells. The apoptosis induction at 24 h, shown in our experiments after UPEB irradiation, also represents a unique finding, since it has been previously shown that the MRC5 cell line can tolerate high doses of irradiation (up to 80 Gy, X-ray) without apoptosis induction [21]. As was suggested by Bluwstein et al. [22], the irradiation of MRC5 cells leads to PKC signaling induction, which in its turn inhibits apoptosis. Whether UPEB irradiation affects the PKC signaling pathway remains to be established.

## 4. Materials and Methods

### 4.1. Cell Culture and Irradiation

The MRC5 (human fetal lung fibroblasts) cell line was maintained in DMEM (Sigma Aldrich, Darmstadt, Germany), supplemented with 10% fetal bovine serum (HyClone, Buckinghamshire, UK), 2 mM L-glutamine (Sigma Aldrich, Darmstadt, Germany), 100 IU/mL penicillin (Sigma Aldrich, Darmstadt, Germany), and 100 μg/mL streptomycin (Sigma Aldrich, Darmstadt, Germany) at 37 °C in 5% CO_2_. Radiation treatment was carried out using an electron beam generated by a laser-driven radiofrequency gun-based linear AREAL accelerator. The characteristics of the AREAL accelerator have been described previously [13]. The parameters of the AREAL laser-generated electron beam are presented in Table 1.

Prior to irradiation, cells were seeded at a density of 0.4 × 10^5^ cells/mL in 2.5 mL of culture medium onto coverslips (SPL Lifesciences, Gyeonggi-do, South Korea) placed inside 35-mm Petri dishes (Corning, New York, NY, USA) and incubated at 37 °C and 5% CO_2_ for 20 h. Directly before irradiation, the Petri dishes were completely filled with culture medium and enclosed with sterile Parafilm to allow for the upright exposure of cell samples at the horizontal beam. For cell irradiation, each sample was placed in a sample holder facing towards the horizontal beam coming from the direction of the vacuum window, thus minimizing the material in front of the cell suspension. Cell samples were placed vertically at the center of a 3 cm × 3 cm area, 1 cm from the beam exit point of the accelerator. The dosimetric measurements were performed with a Faraday cup (commercially available), estimating the integral dose over the pulse. Cells were irradiated on ice [39] at doses of 0.1 Gy (14 electron pulses), 0.5 Gy (70 electron pulses), and 1 Gy (139 electron pulses) with a repetition rate of 2 Hz. A peak dose rate of 1.6 × 10^10^ Gy/s was estimated based on the estimated pulse duration of 4.5 × 10^−13^ s, itself based on the laser pulse length, acceleration process, and electron beam transport. The mean absorbed dose rate of 0.899 ± 0.0036 Gy/min was calculated over the period of irradiation and 1% charge fluctuation and 1% beam energy fluctuation was taken into account. The X-ray reference irradiation was performed for each biological endpoint using a RUM-17 X-ray machine (Mosrentgen, Moscow, Russia; 150 kV, 10 mA, 0.5 mm Cu and 1-mm Al filters, 0.24 Gy/min). Doses were determined with an ionization chamber and by chemical dosimetry. All X-ray irradiation was performed using the same cell culture concentration and Petri dishes that were used for the UPEB irradiation at the same temperature. Mock-irradiated (0 Gy) cells were used as a control.

### 4.2. Apoptosis Analysis

Cell death was estimated 24 h after irradiation using FITC-conjugated Annexin V/7AAD assay (BioLegend, London, UK) by flow cytometry, according to the manufacture’s instruction. Briefly, cells were harvested, washed twice with cold PBS, and resuspended in 1 mL Annexin binding buffer. Afterwards, 100 μL of cell suspension were transferred to a test tube and stained with 5 μL Annexin V-FITC and 10 μL 7AAD. The cells were incubated for 15 min at room temperature in the dark and 400 μL of Annexin binding buffer were added prior to acquisition. Samples were analyzed by BD FACScan flow cytometer (Becton Dickinson, San Jose, CA, USA) and at least 20,000 events were obtained. The data were analyzed using FlowJo version 10.1 (Ashland, OR, USA).

### 4.3. CBMN Assay

Cytokinesis-block micronucleus (CBMN) assay was performed according to the method described by Fenech [40]. After 24 h of irradiation, Cytochalasin B (3 µg/mL, Sigma Aldrich, St. Louis, MO, USA) was added into the cell culture and incubated at 37 °C. After 72 h of incubation, the cells were harvested via trypsinization, washed with PBS, and fixed twice in ethanol/acetic acid (3:1). Fixed cells were smeared on a precleaned microscope slides and air-dried. Staining was performed with Giemsa (10%) for 5–7 min. Scoring of binucleated cells was conducted using a light microscope (HumanScope, Barrie, ON, Canada). A total of 1000 binucleated cells were scored and the frequency of binucleated cells with micronuclei (MN) was determined.

### 4.4. Immunofluorescence Staining

Cells were fixed on coverslips in 4% paraformaldehyde in PBS (pH 7.4) for 20 min at room temperature, followed by two rinses in PBS and permeabilization in 0.3% Triton-X100 (in PBS, pH 7.4), supplemented with 2% bovine serum albumin (BSA) to block nonspecific antibody binding. Cells were incubated for 1 h at room temperature with a primary antibody against γH2AX (dilution 1:200, clone JBW301, Merck-Millipore, Burlington, VT, USA) and p-DNA-PK (dilution 1:200, ab18192, Abcam, Cambridge, MA, USA) diluted in PBS with 1% BSA. After several rinses with PBS, cells were incubated for 1 h with secondary antibodies IgG (H+L) goat anti-mouse (Alexa Fluor 488 conjugated, dilution 1:600; Merck-Millipore, Burlington, VT, USA) and goat anti-rabbit (rhodamine conjugated, dilution 1:400; Merck-Millipore, Burlington, VT, USA) diluted in PBS (pH 7.4) with 1% BSA. Coverslips were then rinsed several times with PBS and mounted on microscope slides with ProLong Gold medium (Life Technologies, Carlsbad, SA, USA) with DAPI. Cells were imaged using a Nikon Eclipse Ni-U microscope (Nikon, Tokyo, Japan) equipped with a ProgRes MFcool camera (Jenoptik AG, Jena, Germany). The filter sets used were UV-2E/C (340–380 nm excitation and 435–485 nm emission), B-2E/C (465–495 nm excitation and 515–555 nm emission), and Y-2E/C (540–580 nm excitation and 600–660 nm emission). At least 300–400 cells were imaged for each data point. Foci were enumerated using FociCounter software (http://focicounter.sourceforge.net/). Approximately 1% of the nuclei were substantially larger than normal (possibly indicating the presence of tetraploid or G2-phase cells), and were not considered for evaluation.

### 4.5. Statistical Analysis

Statistical analyses of the data were conducted using GraphPad Prism 5.01 (GraphPad Software, San Diego, CA, USA). The results are presented as the means of three independent experiments ± standard error. Data were analyzed by repeated measures ANOVA and the differences between groups were determined by Dunn’s post hoc test. *p-*value < 0.05 was considered the statistically significant value.

## 5. Conclusions

This study revealed the different radiobiological effects of UPEB radiation in vitro, in comparison to X-ray radiation. In contrast to X-ray, the UPEB-induced γH2AX foci were characterized by slow elimination kinetics, suggesting the formation of more complex DNA damage. The lower level of genotoxic capacity, as reflected by the slight increase in micronuclei frequency in the case of UPEB irradiation, was shown, and can be explained by the elimination of cells with highly damaged DNA via apoptosis, observed only after UPEB irradiation.

## Figures and Tables

**Table 1:** Characterization of AREAL laser-generated electron beam.

AREAL Beam Parameters		UV Laser Parameters	
Beam charge (pC)	30	Wavelength (nm)	258
Electron energy (MeV)	3.6	Pulse energy (μJ)	200
Pulse duration (fs)	450	Repetition rate (Hz)	2
Pulse repetition rate (Hz)	2	Energy stability	<2%
Beam spot (mm)	15	Beam divergence (mrad)	<0.3
Norm. emittance (mm-mrad)	<0.5	Beam diameter (mm)	2.0
RMS energy spread	<1.5%	-	-
Online dose information	Faraday cup	-	-

## Abbreviations

AREAL	Advanced Research Electron Accelerator Laboratory
BN	Binuclear
BSA	Bovine serum albumin
CBMN	Cytokinesis-block micronucleus
DMEM	Dulbecco’s Modified Eagle Medium
DNA	Deoxyribonucleic acid
DSBs	Double-strand breaks
GHz	Gigahertz
Gy	Gray
LET	Linear energy transfer
MeV	Megaelectronvolt
MN	Micronuclei
PBS	Phosphate-buffered saline
RF	Radio frequency
UPEB	Ultrashort pulsed electron beam
